# Boundaries, Limits, Global Threats – How Can the Impacts of Global Synthetic Pollutants Be Reduced?

**DOI:** 10.1021/acs.est.5c13807

**Published:** 2026-02-02

**Authors:** Martin Scheringer, Hans Peter H. Arp, Ian T. Cousins

**Affiliations:** † Institute of Biogeochemistry and Pollutant Dynamics, ETH Zürich, 8092 Zürich, Switzerland; ‡ Norwegian Geotechnical Institute (NGI), 0484 Oslo, Norway; § Department of Chemistry, Norwegian University of Science and Technology (NTNU), 7491 Trondheim, Norway; ∥ Department of Environmental Science, 7675 Stockholm University, SE-10691 Stockholm, Sweden

**Keywords:** persistence, chlorofluorocarbons, per- and polyfluoroalkyl substances, planetary boundaries, limits to growth

## Abstract

The planetary-scale risks posed by “chemicals of global concern” have deep historical roots that predate the literature on the Planetary Boundaries concept. Two largely separate scientific and regulatory tracks emerged from mid-20th-century research: an atmospheric track (exemplified by chlorofluorocarbons and stratospheric ozone depletion) and an aquatic-terrestrial/ecotoxicological track (exemplified by DDT, PCBs and other bioaccumulative organohalogens). Both tracks produced early warnings, scientific consensus, and eventual multilateral environmental agreements (the Montreal Protocol and Stockholm Convention). In this Perspective, we synthesize the historical evidence, link it to the planetary-boundaries and limits-to-growth narratives, highlight why chemical regulation repeatedly failed to prevent widespread contamination, and propose a set of pragmatic policy instruments, including targeted premarket controls such as the application of the Safe and Sustainable by Design framework, class-based phase-outs to speed up the removal of hazardous substances from the market, and global burden sharing to better manage planetary-scale chemical problems.

## Graphical abstract

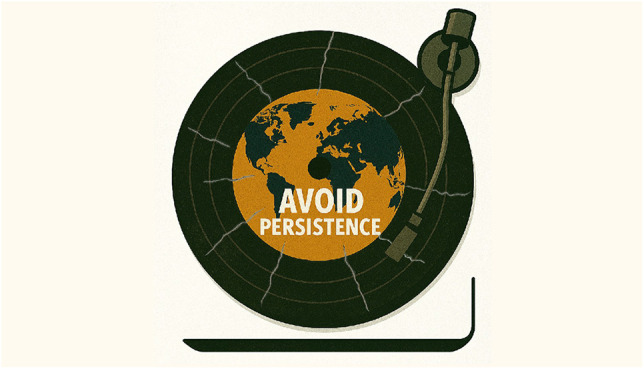

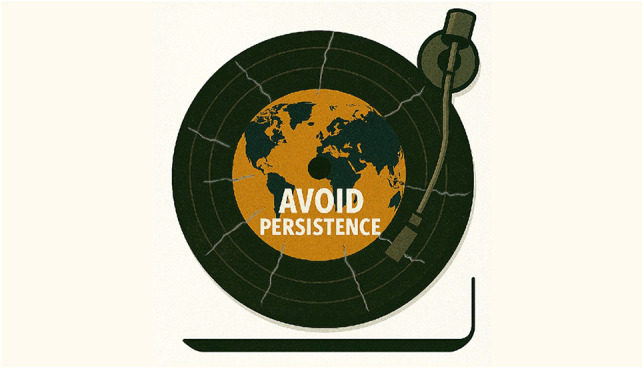

## Introduction

The Planetary Boundaries concept was initially published by Rockström et al. in 2009 and has since then been adapted and modified several times.^1–3^ In the initial publication of the concept, chemicals were covered by an impact called “chemical pollution”, but the extent of this impact was labeled as “not yet quantified”. Steffen et al. (2015)^2^ later changed the label from “chemical pollution” to “novel entities” based on the following definition: “We define novel entities as new substances, new forms of existing substances and modified life-forms that have the potential for unwanted geophysical and/or biological effects” [2, p. 6]. In 2023, Rockström et al. (2023) introduced a new layer with the concept of “safe and just earth system boundaries” (ESBs) in order to address environmental and social impacts in combination.^3^ In their Figure 1, they presented the concept graphically with eight instead of nine anthropogenic impacts; the novel entities were dropped from the figure, but mentioned in the text.

The Planetary Boundaries concept has also been discussed specifically in connection with chemicals by Persson et al. (2013), MacLeod et al. (2014), Diamond et al. (2015), and Persson et al. (2022).^4–7^ Persson et al. (2013) argued that the “novel entities” boundary is not well-defined, and likely represents many distinct threats rather than a single boundary. They identify three simultaneous conditions that a chemical (or mixtures of chemicals) must meet to pose a planetary boundary risk: (1) the chemical shows a previously unknown disruptive effect on human health, ecological health, of earth system processes; (2) the effect is not discovered until the chemical has impacts at a global scale; and (3) the effect (or the underlying exposure as the cause of the effect) is not readily reversible. The paper proposes strategies for proactively identifying such chemicals (for example, assessing long-range transport, persistence, and potential irreversible effects), in order to manage hazards before they cross thresholds and cause planetary-scale harm.^4^

MacLeod et al. (2014) built on Persson et al. by translating their three criteria into chemical profiles based on the chemicals’ effects and environmental exposures. They suggested that prioritizing commercial chemicals against these profiles is feasible, though significant uncertainties and scientific challenges remain.^5^

Diamond et al. (2015) took a different approach from Persson et al. and MacLeod et al. by attempting to quantify a single, global boundary for novel entities, rather than focusing on identifying chemicals (or mixtures) that individually meet planetary-boundary risk criteria. They pointed out that all synthetic chemicals taken together are difficult to characterize as a single Planetary Boundary threat because both exposure and impacts are extremely heterogeneous, often local or regional and diverse in scale, timing, severity, and there is no single response variable that would indicate the overall severity of such a Planetary Boundary threat.^6^ This was echoed by Persson et al. (2022), who investigated and compared several possible control variables to come to the conclusion that humanity already exceeds the planetary boundary for novel entities because the number of chemicals is so large—and still increasing—that it exceeds the scientific and regulatory capacity for assessment and monitoring. Furthermore, they see plastic pollution as a factor that causes large-scale impacts in such a way that earth system functions may be disturbed.^7^ The issue of accumulating plastic pollution was also earlier highlighted as a Planetary Boundary threat, due to a combination of both known and unknown physical and chemical impacts that would be irreversible on a global scale.^8,9^

Against this background, we explore here the historical context of the Planetary Boundaries concept and its roots in earlier warnings of the impacts of global synthetic pollutants and discuss why political action has not effectively addressed these impacts. This is particularly important because the problem of global synthetic pollutants is not new, but originated already 50 or 60 years ago, and was also clearly indicated as a source of concern. We thereby address the question: what makes the problem of accumulating global synthetic pollutants so intractable?

## Threats from Global Synthetic Pollutants: Two Tracks of Research and Regulation

The case has been made that highly persistent chemicals, such as per- and polyfluoroalkyl substances (PFASs), form a Planetary Boundary threat because they fulfill the requirements for such a threat proposed by Persson et al. (2013).^10^ Also chlorofluorocarbons (CFCs) fulfill these requirements. These are cases where the global or planetary scale of the chemical pollution is most obvious. Importantly, the concern about such cases—persistent chemicals causing impacts on the planetary scale, called “global synthetic pollutants” here—was already expressed long before the Planetary Boundary concept was formulated. There are two “tracks” of scientific work originating in the 1960s or even earlier and dealing with global synthetic pollutants: research into volatile organofluorine chemicals, on the one hand, and research into often bioaccumulative organochlorine and organobromine chemicals, on the other hand. These two tracks were mostly separated for many years, also because the paradigmatic substances of each track—chlorofluorocarbons (CFCs) vs dichlorodiphenyltrichloroethane (DDT) and polychlorinated biphenyls (PCBs)—have very different physicochemical properties and environmental impacts, which implied that they fall into largely separate subdisciplines of the environmental sciences.

Track 1, or the “atmospheric track,” focuses on volatile halogenated gases that drive stratospheric ozone depletion, contribute to climate change or pose long-term health risks. Key milestones include Molina and Rowland’s work presenting the ozone-depletion hypothesis,^11^ the measurement of the ozone hole by Farman et al.,^12^ and the Nobel Prize in Chemistry awarded to Crutzen, Molina, and Rowland in 1995. The subsequent CFC replacements (hydrochlorofluorocarbons, HCFCs, and hydrofluorocarbons, HFCs) are powerful greenhouse gases which have contributed to climate change. Hydrofluoroolefins, HFOs, as replacements for HCFCs and HFCs, degrade into trifluoroacetic acid (TFA), a persistent and mobile substance that may cause long-term, hard-to-reverse health and environmental impacts without prompt intervention.^13^ Track 1 is therefore related to three separate planetary boundaries, namely; ozone depletion, global climate change and novel entities.

Track 2, or the “aquatic-terrestrial and ecotoxicological track,” deals with bioaccumulative organochlorine and organobromine compounds, exemplified by DDT, PCBs, and polybrominated diphenyl ethers, PBDEs. Early research by Jensen et al. in 1969 ^14^ and Goldberg in 1975,^15^ along with Great Lakes studies in the 1980s and 1990s^16^ and extensive research into chemical contamination of the Arctic,^17,18^ led to global recognition of persistent organic pollutants (POPs) in the 1990s and ultimately the Stockholm Convention on POPs. The Convention’s POP criteria, where they are set as numerical thresholds for persistence or bioaccumulation, reflect the properties of these organochlorine substances, highlighting their long-term ecological and human health impacts. From 2001 on, also PFASs were increasingly investigated in this track of research, and several PFASs have been included in the Stockholm Convention.

Each of these two tracks represents a large body of productive scientific research that also provided the basis for a corresponding global treaty (multilateral environmental agreement, MEA) addressing the respective chemicals, namely the Montreal Protocol on Ozone-Depleting Substances of 1989, and later the Kigali Amendment on HFCs as greenhouse gases of 2019, and the Stockholm Convention on POPs of 2004 (years are for entry-into-force).

Interestingly, there have been several instances where these two tracks intersected or overlapped. One was in the 1960s and early 1970s, when research into the enzymatic cleavage of carbon–halogen bonds was conducted. Goldman (1969, 1972) summarizes some of this research; several halogenated acetates, propionates, and butyrates were investigated and the removal of the halogens from these substances was explored. Monofluoroacetate was found to be defluorinated by an enzyme called haloacetate halidohydrolase, but trifluoro- as well as trichloroacetate did not show any dehalogenation.^19,20^ The investigations were detailed and technically sophisticated (including, e.g., various stable-isotope investigations with ^18^O and deuterium). The extraordinary stability of the carbon–fluorine bond was pointed out and Goldman draws the conclusion: “The stability of the carbon–fluorine bond together with the increasing use of fluorocarbons in modern life (aerosol propellants, refrigerants, anesthetics, plastics) suggest that compounds containing the carbon–fluorine bond pose at least a potential threat to the environment”. [20, p. 148]

This was an early warning pointing to problems caused by the persistence of organofluorine chemicals. It nearly coincided with the work by Molina and Rowland (1974) presenting the hypothesis that stratospheric ozone is depleted by CFCs. This effect occurs because of the extraordinary stability of CFCs in the troposphere, which enables them to reach the stratosphere, reflecting exactly the concern expressed by Goldman (1972).^20^

Subsequently, a lot of research was carried out to elucidate the mechanisms governing the behavior of CFCs and of a wide range of CFC replacements in the troposphere (see above, track 1). Some of this work was summarized by Schwarzbach^21^ in 1995 and this was another instance where the two tracks of research, atmospheric and aquatic-terrestrial, overlapped. Schwarzbach pointed out that several important CFC replacements are chemically transformed into TFA in the troposphere. TFA is, in contrast to the parent compounds from which it is formed, extremely persistent, is a PFAS, and is deposited with rain to the surface media, where it accumulates. This analysis by Schwarzbach (1995) connects the earlier concerns about persistent chemicals with the current scientific and political discussion about PFASs as chemicals present in all environmental media and human food and drinking water. A more extensive overview of CFCs and their replacement is, e.g., provided by Burkholder et al.^22^

## The Bigger Picture: Limits to Growth

When Goldman expressed concerns about the stability of organofluorine chemicals and Molina and Rowland presented the hypothesis of ozone depletion by CFCs, these were two elements that contributed to a broader discussion. In 1972, the book, The Limits to Growth (LtG),^23^ was published and it presented for the first time a clear expression of planetary limits to both the extraction of resources and the generation of pollution at the global level. The book reached a wide audience beyond the scientific domain, caused an uproar and stimulated a first international, all-encompassing discussion about limits to the modern economy as a whole, and also questioned the associated lifestyle. Chemicals were not considered explicitly in the LtG’s modeling of the global system of economy, population, resource consumption and generation of pollution; pollution was represented by emissions of carbon dioxide. The omission of chemicals as an explicit component of pollution may be explained by a lack of sufficiently comprehensive data on chemical production and emissions in the late 1960s.

However, at the time of LtG, chemicals were already well recognized as a threat to human health and the environment as a consequence of Rachel Carson’s Silent Spring (1962).^24^ Carson also provided some numbers [24, chapter 2]: “new chemicals come from our laboratories in an endless stream; almost five hundred annually find their way into actual use in the United States alone. The figure is staggering and its implications are not easily grasped–500 new chemicals to which the bodies of men and animals are required somehow to adapt each year, chemicals totally outside the limits of biologic experience”.

The LtG modeling of the global system was carried out with a rather simple “world model” called *World3* that contained relevant interactions and feedback loops between the model variables.^23^ The model results were not meant to represent predictions, but illustrated different scenarios of possible developments. The standard run of the model shows a peak of population, industrial output, food production etc. in the first third of the 21st century, followed by a strong decline.

On the one hand, LtG stimulated public concern (and reflections) about the global impacts of the modern extractive economy; it also contributed to social movements searching for new lifestyles, including US president Jimmy Carter’s attempts to save energy; the concept of a “New Age” purported by physicist Fritjof Capra, anthropologist Gregory Bateson and others.^25^ On the other hand, the book and its findings were ridiculed by economists and many politicians and its main findings were considered an insult to the idea of a continuous progress of modern societies. Nevertheless, the concept of (hard) limits had, for the first time after the post-WWII boom, been formulated and substantiated quantitatively. As a new element of the work around LtG, analyses of empirical data corresponding to the parameters of the *World3* model have been published by Turner (2012) and Herrington (2020).^26,27^ These data series covering some 40 years demonstrate empirically that the current pathway of the global economy is close to the standard run of the *World3* model.

LtG and the Planetary Boundary concept represent two complementary (and overlapping) frameworks that both indicate that the current trend of exponential growth with its associated resource extraction and waste generation (chemicals released to the environment after use are a form of waste) increasingly causes detrimental impacts at the global scale.^27,28^ The chemical industry causes 18% of all industrial-sector CO_2_ emissions and 5% of the global combustion-related CO_2_ emissions,^29^ which indicates a tight connection between chemical pollution and greenhouse gas emissions. Both concepts, LtG and Planetary Boundaries, emphasize that many impacts of the global economy (boundary threats) are mutually dependent and may reinforce one another and that the underlying drivers cannot be managed individually. Moreover, many Planetary Boundary threats are related to large-scale, irreversible changes to the chemistry of the atmosphere and the aquatic and terrestrial environments: ozone-depleting substances; CO_2_ and other greenhouse gases; atmospheric aerosol formation from combustion processes; CO_2_ leading to ocean acidification; mobilization of huge amounts of nitrogen and phosphorus; and huge emissions of global synthetic pollutants.

Specifically in the context of synthetic chemicals, both global synthetic pollutants with chemical-specific impacts such as CFCs, PFASs etc. as well as the global production of chemicals as a massively fossil-fuel dependent process contribute to Planetary Boundary threats.

## Early Warnings, but No Lessons Learned

In 1992, the Rio Declaration on Environment and Development was published.^30^ In its Principle 15 it calls for a precautionary approach to “threats of serious or irreversible damage”. This reflects an international recognition of long-term threats and the need to counter these threats before their consequences have fully manifested themselves.

The European Environment Agency (EEA) investigated several chemical pollution issues in two extensive reports on “Late Lessons from Early Warnings” ^31,32^ and analyzed the history of these pollution issues from early warnings to manifest impacts and (often late) action. Foss Hansen et al. (2024) applied the framework of the EEA reports to the PFAS case and explored reasons for the lack of effective action in spite of many strong indications that the PFAS problem was serious and spiraling out of control.^33^ Specifically, they identified four factors: (i) for a long time authorities relied on voluntary measures to be taken by the industry; (ii) there were no grouping approaches in the set of regulatory measures and no accepted way of extrapolating from a small number of well-investigated chemicals to a larger group; (iii) there is a strong focus on “significant risks”, and these manifest themselves only after long periods of extensive use of a chemical; (iv) PFASs have become pervasive in a large number of applications and this constitutes a technical lock-in.

Taken together, these four factors provide a plausible explanation of the lack of action, not only in the case of PFASs but also for other global synthetic pollutants, in particular plastics.^8,9,34^ “Significant risks” were not yet visible in the early stages of using a particular type of chemical and applications became more and broader. Only when the chemical had been used and emitted in large amounts (and, thereby, had become an important component of various technologies), the impacts became sufficiently strong and visible, but the lock-in then made action slow and ineffective.

The crucial follow-up question is whether the concept of safe and just ESBs can help to overcome these hindrances. The scientific evidence has for long been available for several groups of global synthetic pollutants (CFCs, POPs, plastics, PFASs) that have caused serious global impacts.^35^ Therefore, it is not so much the lack of scientific knowledge that blocks the way to solutions; what may be more effective (and is still underrepresented in chemicals management) is indicated by the justice component of just ESBs: chemical pollution, in particular by global synthetic pollutants, causes unjust distributions of benefits and burdens.^36^ This aspect of environmental justice has so far not been fully exploited and should be given much stronger emphasis. Gupta et al. (2025) extensively discuss the introduction of just ESBs, but entirely leave out pollution by synthetic chemicals.^37^ We maintain that also pollution by synthetic chemicals contributes massively to an unjust distribution of impacts on human health and the environment and that it should be included explicitly in the concept of just ESBs, which we recommend as an important task for future work on the ESB concept.

## Implications

The Planetary Boundary concept is a useful framework for visualizing, quantifying and communicating global threats. It also shows that several threats are interconnected and that many are caused by anthropogenic emissions of various types of chemicals, from nitrogen and phosphorus to carbon dioxide, ozone-depleting substances, and the large bulk of other chemicals in the “novel entity” group. Within the group of novel entities, it is most effective to specify planetary boundaries for global synthetic pollutants such as long-chain perfluoroalkyl acids or trifluoroacetic acid because emission sources, environmental fate and adverse effects are relatively well-defined and globally relevant, which makes the point of a Planetary Boundary threat plausible. This is also the logic according to which Rockström et al. (2009) singled out CFCs and assigned them a separate Planetary Boundary threat although they are global synthetic pollutants like PFASs or plastics. New Planetary Boundary threats can be defined for PFOA,^10^ where the global threat reflects the fact that PFOA is a human carcinogen, and for TFA, where the global threat derives from TFA possibly being toxic for reproduction.^38^ This is also in line with Persson et al. (2013), who state “that “chemical pollution” is not a single category in the Planetary Boundary framework”.^4^

Many insights into the global threat from persistent, globally present chemicals were expressed earlier, even as early as the 1960s and 1970s.^11,14,20,24,39^ With LtG, there was also an early framework addressing the broader problem of excessive resource consumption and waste generation. Given that the impacts and related concerns have only been growing since the 1970s, it is necessary to focus even more strongly on the global threats from global synthetic pollutants. Specifically, we see several lessons to be learned and related opportunities for action:

First, in connection with Planetary Boundary threats from synthetic chemicals, the property that stands out is persistence. Environmental persistence can facilitate the global distribution of chemicals (and the associated long-term and large-scale human exposure and, then, associated effects). Accordingly, persistent chemicals need to be strictly avoided wherever possible. This does not imply that nonpersistent, but highly toxic chemicals are not a problem as well, but the problem is of a different type and structure and requires a different management approach. Often, the adverse effects caused by a chemical are identified and understood only after many years of use. For example, PFOA was officially identified as a human carcinogen only in 2024, 75 years after its introduction into the market.^40^ Chemicals that are not particularly toxic but highly persistent need to be given higher attention and should be avoided even if their toxicity is not pronounced. Plastics are a particularly striking example of this problem. Many plastics are generally considered as nontoxic, but are highly persistent and their persistence has led to the buildup of an enormous amount of waste plastic in the global ecosystem with a wide range of massive impacts, including impacts from nonpolymeric plastic additives^9,41,42^ and from the slow release of microplastics, nanoplastics and diverse leachates from weathering.^43^

Second, to reduce the overwhelming burden on regulatory agencies that results from the enormous number of chemicals to be assessed, grouping approaches should be used more frequently, for example for PFASs^44^ and bisphenols,^45^ but possibly also other groups of similar substances. A main advantage of chemical grouping is that a restricted or banned chemical cannot be replaced by a similar chemical from the same group.^46^ A second conclusion from the huge number of chemicals on the market is that the number of chemical uses needs to be reduced,^47,48^ as it is unlikely that the capacity of regulatory bodies will be increased substantially in the near future.

A third lesson to be learned is that better and more effective premarket assessments of new chemicals are needed. The sheer number of chemicals entering the market without sufficient assessment is a problem. The concept of Safe and Sustainable by Design^49^ offers methods for a more extensive and systematic assessment of new chemical products and provides guidance on the selection of less problematic substances.

A fourth lesson is that extensive use of a chemical leads to lock-ins because many users rely on the chemical.^48^ Even if unwanted effects of such a chemical become known, producers and users are reluctant to start a transition to less problematic alternatives because this may be associated with substantial effort and cost. Bisphenols and PFASs are examples here. The development and supply of alternatives to the locked-in chemicals should be supported, instead of just a focus on the demand for “established” chemicals. Alternatives can be technological or material solutions rather than simply drop-in chemical substitutes. Technological alternatives include different designs of a process or a technical installation so that the need for a certain type of chemical may disappear. An example of a technological alternative is applying underpressure in baths for chrome plating, which makes it possible to work without PFOS as a mist suppressant. Material alternatives can serve a similar function and eliminate the use of a hazardous chemical, e.g., using fiberglass insulation in place of foam products that require fluorinated gases as blowing agents. In addition to using alternatives, reducing demand by demonstrating that certain product functions are nonessential, e.g., stain repellency in certain consumer textiles where stain repellency is not needed, can be a more effective approach to eliminating the need for problematic chemicals.^50^

Given all this, a sufficient body of scientific knowledge describing the problem of Planetary Boundary threats from chemicals exists. The bottleneck is the steps toward more effective action to combat and avoid such problems. This is not a scientific problem, but a problem of the political process using (or not using) scientific information. Scientists can and should also be present in this process, but this requires suitable formats, i.e. science–policy interface bodies—from the regional to the national and international level—that enable a structured and continuous transfer of scientific knowledge into the political process. One example is the recent effort of the Swiss Academy of natural sciences in the discussion of PFASs in the Swiss parliament, which included a meeting of Members of Parliament with scientists, the preparation of a PFAS fact sheet for a general audience, including Members of Parliament, and the publication of an opinion piece by scientists directly targeted to Members of Parliament for their parliamentary discussion about PFASs.^51,52^ Another example is California’s Office of Environmental Health Hazard Assessment (OEHHA), which facilitates scientific input into the State’s regulation.^53^ Such interactions should not be the exception, but become the norm, in order to raise awareness and action against the irreversible threat of accumulating global synthetic pollutants. Without systemic reform, scientists risk sounding like a broken record—repeating the same messages across generations (e.g., “avoid highly persistent substances”), while inaction perpetuates global contamination.
